# Potential use of beet-pulp concentrate supplementation in athletic horse

**DOI:** 10.1016/j.heliyon.2024.e40961

**Published:** 2024-12-10

**Authors:** Luzilene Araujo de Souza, Monica Miranda Hunka, Sigismundo Fassbender de Rezende Júnior, Carolina Jones Ferreira Lima da Silva, Helena Emília Cavalcanti da Costa Cordeiro Manso, Joana Simões, Clarisse Simões Coelho, Francesco Fazio, Francesca Aragona, Hélio Cordeiro Manso Filho

**Affiliations:** a Center for Equine Research and Laboratory of Molecular Biology Applied to Animal Production (BIOPA), Department of Animal Science, Federal Rural University of Pernambuco, Rua Dom Manuel Medeiros, 52171-900, Recife, Pernambuco, Brazil; b CECAV, Faculty of Veterinary Medicine, Lusofona University (ULHT), Campo Grande 376, 1749-024, Lisbon, Portugal; c CIISA-Centre for Interdisciplinary Research in Animal Health, Faculty of Veterinary Medicine, University of Lisbon, 1300-477, Lisbon, Portugal; d Associate Laboratory for Animal and Veterinary Science (AL4AnimalS), Faculty of Veterinary Medicine, University of Lisbon, 1300-477, Lisbon, Portugal; e Mediterranean Institute for Agriculture, Environment and Development, Universidade de Évora, Évora, Portugal; f Department of Veterinary Sciences, University of Messina, Italy

**Keywords:** Cortisol, Athletic horses, NEFA, Highly digestible fibers, Supplementation

## Abstract

The aim of this two-phase study was to evaluate the use of beet pulp concentrate (BPC) in athletic horses. On the first trial the effects of supplementation with 820g of BPC for 12 weeks were assessed and a crossover postprandial curves of blood biomarkers were determined in five adult Arabian mares. Differences were found in T-chol, HDL-chol, TPP and MCHC (p < 0.05). Postprandial curves varied among challenges (p < 0.05) for glucose, insulin, and hematocrit. These results confirmed that BPC supplementation led to changes in blood biomarkers, without inducing major metabolic disruption. The aim of the second trial was to evaluate effects of resistance training using a horse walker in eight yearling gaited foals fed with BPC (16 %) for 12 weeks. Blood samples were collected before and at 30, 60 and 90 days of training and results demonstrated the highest level of [NEFA] and [triglycerides] at end of experimental period (p < 0.05) and increased of growth hormone after exercise sections after 60 days (p < 0.05). Also, [MCHC] and [Hb] increased 30 days after beginning of training program (p < 0.05), maintaining until the end of trial period. Association of the training program with highly digestible fiber as BPC possibly led to a greater availability of NEFA and hCG certainly helping the physical conditioning of these young animals.

## 1 Introduction

The concentrate feeds used regularly for sport horses contain large amounts of non-fibrous carbohydrates, such as starch, which may compromise their health and affect their digestive process [1,2]. However, in recent years, this problem was addressed by adding highly digestible fibers such as beet pulp, alfalfa, and soybean hulls, which stimulates the production of short-chain fatty acids (SCFA) by intestinal microbiome, thereby contributing to microbiota diversity and animal health [3,4]. Also, these special fibers contain more digestible fibers rich in pectin [1,5,6] and their inclusion can increase chewing time [6], which is beneficial for the digestive process, especially in horses that are under regular and intense training programs and frequently do not receive enough food to maintain a healthy gastroin-testinal function. Horses are herbivorous animals, and their diet must be rich in fiber, for athletic animals. Diets rich in fiber, fat or carbohydrates have a different impact on the equine gastrointestinal tract. However, when horses consume more digestible fiber, their microbiota presents greater diversity and richness, contributing to the production and absorption of SCFA [7,8]. Furthermore, it has been proved that horses with diets rich in fiber, especially digestive ones, have similar performance to animals with traditional diets rich in soluble carbohydrates [9,10] but may have more fluid stored on internal compartment, like colon and cecum, which is important for equine athletes that compete in endurance and four-beat gaited events [[11], [12], [13]]. Recently it has been shown that when horses receive diets rich in carbohydrates, compared to diets rich in digestible fiber, there is a greater disruption in the fermentation processes throughout the entire gastrointestinal tract [[14], [15], [16], [17]]. This can lead not only to digestive diseases, but also to physiological and behavioral changes in horses fed these diets. Previous experiments have shown that diets with more fiber increased chewing time and saliva production, which was associated with the reduction of oral behaviors and improves in horse behavior [7,8,15,18]. So, inclusion of high-quality fibers, such as alfalfa and beet pulp, may contribute to the health and well-being of athletic horses. The effects of highly digestible fibers on the digestive process of horses and the response of blood biomarkers to the inclusion of such supplements must be well characterized for equines with different ages, particularly for sports animals, given their high energy requirements [19]. Proper nutritional programs for athletic horses can have a positive impact on the performance of upcoming athletes [20]. In order to test the hypothesis that supplementation with beet-pulp concentrate improves the availability of energy sources, bringing beneficial effects for young and adult horses without impacting their health, a study was developed in two phases: (1) testing the effects of a 12 week-supplementation protocol with 820g of beet pulp concentrate (BPC) on the blood biomarkers of adult broodmares, proofing the safety of this kind of concentrate for horses; then (2) evaluate the impact of an aerobic resistance training program on an automatic exerciser for yearling foals, supplemented with a concentrate rich in digestible fiber (BPC).

## 2 Materials and methods

### 2.1 Trial 1: supplementation during 90 days with BPC in broodmares

The experiment was carried out in the dry summer, and as a result the amount of pasture was reduced and therefore the animals are supplemented with hay. These animals consumed around 10 kg of hay per day and little grass where they were kept free. These animals were not breeding stock, but rather maintenance animals, meaning they were adult horses that were not engaged in stimulated exercise or in a state of pregnancy/lactation. The cross-over experiment involved five healthy, purebred Arabian mares with an average age of 14 ± 3 years, average body mass of 367 kg, and body condition score 5 (1/9 scale) [21]. The enrolled mares were clinically healthy, and they belonged to the Department of Zootechnics of the Federal Rural University of Pernambuco (UFRPE) in Recife-PE, Brazil. These animals were kept on a paddock (2.5 ha) with native pasture grasses and were continuously moni-tored to ensure their health and well-being during the experimental period [22,23]. In animals under maintenance, the impact of beet pulp on health was tested as it is not a product regularly used in South America, particularly in a farm setting. The animals had not been given any concentrate feed in the four weeks prior to the experimental protocol. They had free access the pasture, spending over 22 h a day foraging freely, and to Tifton hay (Cynodon nlemfuensis) and mineral salt for horses (CoEqui, Tortuga, Brazil), ad libitum. The supplementation program followed the National Research Council [12] guidelines for the maintenance of adult horses weighing 400 kg, with 50 % of their energy needs being supplied by the beet-pulp concentrate (BPC) (Gua-biTech BEET, Guabi Nutrição Animal, Brazil), which corresponded to 860.0g of this product (Table 1). Thus, for 12 weeks, all mares were individually fed in their boxes, twice daily (6AM and 4PM). Immediately before and after the 12-week supplementation period a pre-and post-supplementation postprandial curve was performed using a crossover model (5 animals and 3 treatments).

**Table 1:** Content and bromatological analysis, in dry matter, of concentrate rich in digestible fiber.

Component	Assured content	Bromatological analysis
Crude protein	12 % minimum	13.94 %
Ether extract	11 % minimum	12.18 %
Crude fiber	10 % maximum	–
Neutral detergent fiber	–	28.35 %
Acid detergent fiber	–	12.48 %
Mineral matter	10 % maximum	7.79 %
Digestible energy	4.06 Mcal/kg	–

Blood samples of all mares were collected at 15-day intervals to evaluate the effects of a 12 week-supplementation protocol with 820g of beet pulp concentrate (BPC), starting on the first day of the trial (in a total of 7 samples), with the horses fasting before sampling. Samples were obtained by jugular venipuncture in three different tubes: with EDTA (for blood cell count), with sodium fluoride (for glucose analysis) and without anticoagulants (for serum biochemical analysis) to assess hematological biomarkers. In addition to the evaluations over the 90 days, the postprandial curves were evaluated using a crossover model, before and after the 90 days, to characterize the glycemic index of the experimental concentrate. For this, corn was used as a control (Control) as it is the most used grain in the region, and two levels of supplementation of the experimental diet (minimum or BPC-1 and maximum or BPC-2) for animals in maintenance indicated in the NRC (2007). These post-prandial curves were used to identify possible adaptations over the 90 days and the glycemic index of the experimental feed before and after the 90 days of supplementation, ensuring the safety of using this product for experimental animals. For that, blood samples were collected at rest (after overnight fastening) and after 30, 60, 90, 120, 180 and 240 min of feeding with three different concentrates (860.0g of whole grain corn, control group; 820.0g of beet pulp concentrate, BPC-1; and 1640 g of beet pulp concentrate, BPC-2), with an interval of five days of washout between them. Isocaloric supplementation was adopted for the Control and BPC-1 treatments during the postprandial curves. Two level of BPC challenges reflect 50 % of NRC [24] recommendation for horses at maintenance, lower (BPC- 1) and higher (BPC-2) levels. Blood samples collected in EDTA tubes were immediately taken to the laboratory for hematological analysis (pocH-100iV Diff hematology analyzer, Sysmex do Brasil, São Paulo, Brazil). This device was used to determine the red blood cell count (RBC), hemoglobin concentration, hematocrit, mean corpuscular volume (MCV), mean corpuscular hemoglobin concentration (MCHC), red blood cell distribution width (RDW)-standard deviation (RDW-SD), RDW- coefficient of variation (RDW-CV), platelets and total leukocyte count. Plasma from these samples was used to determine total plasma protein (TPP) by refractometry. Samples with sodium fluoride and those collected without anticoagulants were centrifuged for 10 min at 3500 rpm for plasma and serum separation, respectively. Plasma was used for glucose determination; serum samples were frozen (−20 °C) for later determination of insulin, total cholesterol (T-chol), HDL cholesterol (HDL-chol), LDL cholesterol (LDL-chol), and triglycerides. Commercial kits were used in a semi-automatic biochemical analyzer (Doles D250, Goiânia, Brazil). In addition, serum insulin concentration was determined by enzyme-linked immunosorbent assay (ELISA) (Mindray MR-96A microplate reader, Bioclin, Belo Horizonte, Brazil). All the analyses were performed in duplicate.

### 2.2 Trial 2: yearling foals exercise and supplementation program

Eight (n = 8) clinically healthy, Mangalarga Marchador, yearlings (12 months old) were enrolled for this trial. The yearlings (5 females and 3 males) had an average weight of 240 kg, a body score of 5.5/9 [21], and were kept in a 6 ha dry lot in Brazil (08o 12′04”S; 35° 33′53”W). During the trial all animals were continuously monitored to ensure welfare [22,25]. For 12 weeks all yearlings were individually fed a combination of two commercially available concentrate feeds, 750g/animal of 16 % BPC plus 100g/animal of a concentrate with 18 % P.B.; 7.5 % E.E.; 11 % F.B.; 11 % M.M.; 3,600 Kcal D.E., twice a day (6AM and 4PM). The latter was included in the yearlings' diet to ensure that the protein requirements recommended by the NRC [24] for these young animals were met (estimated for an adult weight of 400 kg). Additionally, water, salt and corn silage were provided ad libitum. Both feeds covered 50 % of the energy requirements of these horses with the remaining energy being supplied by the forage. In growing horses and performing exercises as indicated, but already understanding a little about the impact of beet pulp on health. This is the distinguishing feature of the experiment, as young animals regularly remain on pastures and do not exercise on equipment with an automatic exerciser. This process impacts the release of hormones, such as GH, and can impact the availability of energy for resistance exercises, such as NEFA. Supplementation with silage, despite being ad libitum, was provided in places where animals were individualized, and at the time of year there was almost no pasture due to the dry summer in the region, which is why we called the 'dry lot' serving as a place of freedom for the foals and thus ensuring their well-being.

The exercise program consisted of a 12-week's training using a semi-automatic circular exerciser for 4 horses (Equiboard®, Jaguariúna, Brazil), with progressive exercise speed and time increase every two weeks (Table 2). The yearling spent the remainder of the time in the dry lot.

**Table 2:** Speed and duration of exercise on the semi-automatic exerciser of the yearlings during the 12-week training protocol.

Week	Speed (m/s)	Time (min)
1st and 2nd	1.67	60
3rd and 4th	1.94	60
5th and 6th	1.67	80

Blood samples were collected from the jugular vein and stored in EDTA tubes and tubes without an additive for complete blood count (CBC) and serum biochemistry analysis. Samples were collected at rest, pre-supplementation and training program and after 8h of fasting, and at days 30 (P1), 60 (P2) and 90 (P3) of the training. At P1, P2 and P3 samples were collected at rest after an 8h fast (A) and immediately after the exercise (B). The protocol used for the CBC, T-chol, HDL-chol, LDL-chol and triglycerides analysis was the same has previously described in Trial 1. Additionally, serum analysis of non-esterified fatty acids (NEFA) was performed, and cortisol and human growth hormone (hGH) levels were assessed with a semi-automated ELISA analyzer (Bioclin Mindray MR-96A, Minas Gerais, Brazil). The body score is used to monitor the animals' nutritional programs. In both groups, the body score did not change over the 90 days. In the first experiment, the subjects were all adult maintenance females, in the second experiment, the animals had not yet reached puberty and were the same age. Furthermore, all animals were in the same environment, separated by sex, and therefore evaluated together. The absence of cross-over in this experiment is insufficient number of animals for this type of experimentation in the place where the animals were housed. Adult horses have not been used because the main objective was to combine the use of beet pulp, which is not common in South America, with exercise in young animals aged up to 12 months. Regarding the growth rate of animals of these breeds in semi-extensive systems, that is, most of the day in the field and only being supplemented at times of the year, when necessary, normally in the dry summer, numerous scientific articles have demonstrated the typical curves of the breed and its growth speed, which is not as high as other breeds such as quarter horses or European saddle breeds.

### 2.3 Statistical analysis

The statistical analysis of both trials was performed using SigmaPlot 13.0 software (Systat Software Inc., San Jose-CA, USA). For the Trial-1, the influence of supplementation on biomarkers was evaluated using one-way ANOVA (time) for repeated measures and Tukey test. Results obtained from postprandial curves (before and after supplementation period) were subjected to two-way ANOVA (treatment and time/phases) for repeated measures and Tukey test. The results of Trial-2 were analyzed using one-way ANOVA and the Tuckey test, in order to assess the influence of the training program and detect possible differences between days. A significance level of 5 % was set for all analyses and the results were expressed as means ± mean standard error.

## 3 Results

### 3.1 Trial 1

All animals consumed the supplied feed within a period of 10–15 min and no clinical nor behavioral changes were observed during the experimental protocol. During the 12-week experimental period, variations in the concentrations of T-chol, HDL-chol, TPP and MCHC (p < 0.05) were detected, reaching their highest levels at the end of the supplementation period (Table 3, Table 4). The remaining biomarkers showed no significant variations throughout the trial.

**Table 3:** Blood biomarkers (means and average standard error) at 2-week intervals during 12 weeks of supplementation with 820.0 g of concentrate rich in digestible fiber in adult mares.

Biomarker		Experimental phase						P value
	Pre-sup-plementation (n = 5)	2nd week (n = 5)	4th week (n = 5)	6th week (n = 5)	8th week (n = 5)	10th week (n = 5)	12th week. (n = 5)	
Glucose, mmol/L	6.23 ± 0.43	7.15 ± 0.27	7.65 ± 0.19	7.52 ± 0.22	6.97 ± 0.26	6.95 ± 0.07	6.56 ± 0.52	ns
Insulin, pmol/L	50.98 ± 12.01	38.48 ± 4.17	31.60 ± 5.14	30.97 ± 5.14	28.20 ± 5.28	28.89 ± 4.31	33.82 ± 6.60	ns
T-chol, mmol/L	2.06 ± 0.07^**b**^	3.13 ± 0.21^**a**^	3.48 ± 0.31^**a**^	3.57 ± 0.35^**a**^	3.66 ± 0.26^**a**^	3.67 ± 0.46^**a**^	3.28 ± 0.29^**ab**^	= 0.010
HDL-chol, mmol/L	1.04 ± 0.11^**b**^	1.90 ± 0.21^**a**^	1.75 ± 0.11^**a**^	1.95 ± 0.10^**a**^	2.05 ± 0.08^**a**^	2.00 ± 0.19^**a**^	2.05 ± 0.18^**a**^	<0.001
LDL-chol, mmol/L	1.02 ± 0.13	1.23 ± 0.18	1.48 ± 0.17	1.02 ± 0.26	1.61 ± 0.23	1.39 ± 0.31	1.12 ± 0.18	ns
Triglycerides, mmol/L	0.53 ± 0.05	0.45 ± 0.02	0.46 ± 0.03	0.49 ± 0.05	0.44 ± 0.04	0.54 ± 0.05	0.44 ± 0.07	ns
TPP, g/L	80.80 ± 2.30^**b**^	93.60 ± 1.80^**ab**^	94.30 ± 1.90^**ab**^	94.90 ± 7.80^**ab**^	98.30 ± 1.00^**a**^	101.50 ± 3.20^**a**^	98.50 ± 4.50^**a**^	<0.022

Note: ns: non-significative; ∗820g BPC/animal/2 times a day; T-chol: total cholesterol; HDL-chol: HDL cholesterol; LDL-chol: LDL cholesterol; TPP: total plasma protein.

**Table 4:** Blood cell count (means and average standard error) at 2-week intervals during 12 weeks of supplementation with 820.0 g of concentrate rich in digestible fiber in adult mares.

Biomarker		Experimental phase						P value
	Before sup-plementation	2nd week	4th week	6th week	8th week	10th week	12th week	
Erythrocyte count, × 10^6^/μl	6.91 ± 0.37	7.63 ± 0.38	6.98 ± 0.23	6.73 ± 0.16	7.62 ± 0.30	7.56 ± 0.27	7.12 ± 0.25	ns
Hgb, g/dL	10.82 ± 0.43	11.98 ± 0.42	12.60 ± 0.70	10.66 ± 0.43	12.14 ± 0.63	12.12 ± 0.48	15.82 ± 2.62	ns
Hct, %	32.28 ± 1.37	35.38 ± 1.35	30.68 ± 0.96	29.70 ± 1.19	33.62 ± 1.63	33.20 ± 1.04	32.33 ± 2.18	ns
MCV, fl	46.84 ± 0.85	46.50 ± 0.81	44.00 ± 0.85	44.06 ± 0.83	44.08 ± 0.81	43.98 ± 0.92	43.88 ± 0.72	ns
MCHC, g/dL	33.54 ± 0.25^c^	33.88 ± 0.34^bc^	37.10 ± 0.89^a^	35.90 ± 0.27^a^	36.08 ± 0.32^a^	36.46 ± 0.41^a^	35.90 ± 0.37^ab^	<0.001
RDW-SD, fl	36.42 ± 0.30	36.36 ± 0.22	35.02 ± 0.28	35.20 ± 0.40	35.42 ± 0.50	35.02 ± 0.55	34.48 ± 0.91	ns
RDW-CV, %	19.20 ± 0.32	19.06 ± 0.41	19.76 ± 0.43	19.68 ± 0.36	19.82 ± 0.34	19.76 ± 0.37	19.94 ± 0.45	ns
Platelets, × 10^3^/μl	124.80 ± 26.65	169.00 ± 20.16	194.40 ± 15.40	193.40 ± 15.44	209.40 ± 22.12	202.20 ± 22.53	160.00 ± 31.52	ns
Leukocytes, × 10^3^/μl	7.26 ± 0.51	7.64 ± 0.41	8.42 ± 0.67	7.46 ± 0.61	7.36 ± 0.62	8.16 ± 0.78	7.16 ± 1.13	ns
Lymphocytes, × 10^3^/μl	2.54 ± 0.32	2.44 ± 0.20	2.32 ± 0.20	2.04 ± 0.33	2.38 ± 0.20	2.48 ± 0.27	2.20 ± 0.40	ns
Other cells, × 10^3^/μl	3.60 ± 0.62	5.20 ± 0.52	6.10 ± 0.68	5.42 ± 0.61	4.98 ± 0.79	5.68 ± 0.95	4.96 ± 1.03	ns

Note: NS: non-significant.

∗820g BPC/animal/2 times a day; Hgb: hemoglobin; Hct: hematocrit; MCV: mean corpuscular volume; MCHC: mean corpuscular hemoglobin concentration; RDW: red blood cell distribution width; SD: standard deviation; CV: coefficient of variation; Others cells: sum of eosinophils, plus basophils and monocytes.

**Fig. 1:**
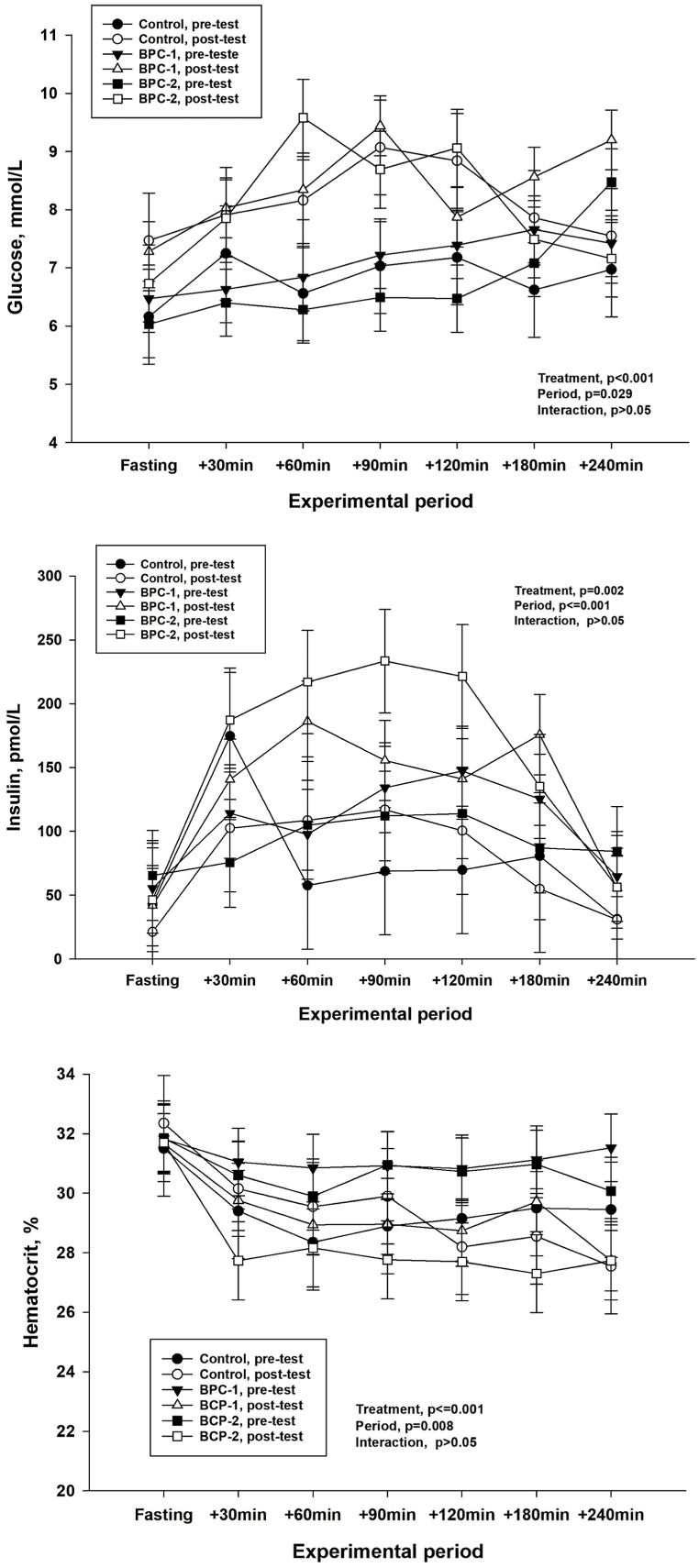
Glucose (A), insulin (B) and hematocrit (C) levels in horses, as a function of treatments during fasting and on postprandi- 43 al curves.

Only the concentrations of glucose and insulin and the percentage of hematocrit varied with time and between treatments in the blood samples collected to determine the postprandial curves before and after the 90 days of supplementation (p < 0.05) (Fig. 1A, B and 1C), but without significant interaction. In addition, mean concentrations recorded in the postprandial curves of glucose, insulin, T-chole, HDL-chole, LDL-chole, triglycerides and hematocrit changed after supplementation (p < 0.05) (Table 5).

**Table 5:** Two-way ANOVA results for mean concentrations in the postprandial curves of the biomarkers, before and after 90 days of supplementation with the experimental concentrate rich in digestible fiber in adult mares.

Biomarker	Experimental phase						p values
	Before supplementation			After supplementation			
	Control (n = 5)	BPC-1 (n = 5)	BPC-2 (n = 5)	Control (n = 5)	BPC-1 (n = 5)	BPC-2 (n = 5)	
**Glucose, mmol/L**	6.82 ± 0.31^**c**^	7.09 ± 0.22^**bc**^	6.75 ± 0.2^**c**^	8.12 ± 0.31^**ab**^	8.39 ± 0.19^**a**^	8.08 ± 0.25^**ab**^	<0.001
**Insulin, pmol/L**	75.08 ± 18.82^**b**^	105.49 ± 13.33^**b**^	91.88 ± 13.33^**b**^	76.46 ± 18.82^**ab**^	128.00 ± 11.88^**ab**^	156.68 ± 15.35^**a**^	<0.001
**T-chol, mmol/L**	2.35 ± 0.15^**b**^	2.14 ± 0.11^**b**^	2.49 ± 0.11^**b**^	3.77 ± 0.15^**a**^	3.35 ± 0.10^**a**^	3.49 ± 0.13^**a**^	<0.001
**HDL-chol, mmol/L**	0.96 ± 0.09^**b**^	1.02 ± 0.07^**b**^	1.00 ± 0.07^**b**^	1.97 ± 0.09^**a**^	1.85 ± 0.06^**a**^	1.86 ± 0.08^**a**^	<0.001
**LDL-chol, mmol/L**	1.39 ± 0.14^**ab**^	1.13 ± 0.10^**b**^	1.49 ± 0.10^**ab**^	1.80 ± 0.14^**a**^	1.56 ± 0.09^**a**^	1.63 ± 0.11^**a**^	= 0.002
**Triglycerides, mmol/L**	0.56 ± 0.03^**a**^	0.49 ± 0.02^**ab**^	0.44 ± 0.02^**bc**^	0.38 ± 0.03^**bc**^	0.36 ± 0.02^**c**^	0.37 ± 0.03^**c**^	<0.001
**Hematocrit, %**	29.46 ± 0.61^**abc**^	31.16 ± 0.43^**a**^	30.73 ± 0.43^**ab**^	29.46 ± 0.6^**abc**^	29.36 ± 0.38^**bc**^	28.30 ± 0.50^**c**^	<0.001
**TPP, g/L**	8.46 ± 0.34	8.61 ± 0.24	9.11 ± 0.24	8.58 ± 0.34	8.54 ± 0.22	8.66 ± 0.28	ns

Note: Different letters in the same line denote significant differences by Tukey test (p < 0.05). Control: 860g of whole grain corn; BPC-1: 820g of commercial concentrate with beet pulp; BPC-2: 1,640g of commercial concentrate with beet pulp; T-chol: total cholesterol; HDL-chol: HDL cholesterol; LDL-chol: LDL cholesterol; TPP: total plasma protein.

### 3.2 Trial 2

The results indicated that significant changes occurred in the studied biomarkers (hCG, NEFA, TG, RBC, Hb, Htc, MCHC, RDW-CV, Platelets) (Table 6, Table 7). The concentrations of NEFA and triglycerides increased at the end of the experimental period of supplementation (p < 0.05), and differences were also observed in the concentration of hGH from the 60d of training, when comparing the blood samples obtained in the pre- and post-exercise of the same phase (p < 0.05). The highest concentration of red blood cells and of hematocrit occurred in the pre-test (p < 0.05), while the concentrations of hemoglobin and MCHC increased 30 days after the beginning of the program (p < 0.05), remaining high until the end of the experiment. The other parameters did not present statistical difference (p > 0.05). It should also be noted that the animals did not show any signs of fatigue and/or clinical illness during the experimental period. Also, no bone or joint lesions were identified in the experimental animals over the 12 weeks.

**Table 6:** Biomarkers of fat metabolism, cortisol and growth hormone in yearling foals of the Mangalarga Marchador breed submitted to a progressive exercise protocol in a semi-automatic exerciser, supplemented with beet-pulp concentrate.

Biomarker	Experimental phase							P value
	Pre-test	30d^a^		60d^b^		90d^c^		
		Pre-exercise	Post-exercise	Pre-exercise	Post-exercise	Pre-exercise	Post-exercise	
**hGH, μIU/mL**	0.51 ± 0.02^**ab**^	0.45 ± 0.03^**b**^	0.50 ± 0.03^**ab**^	0.45 ± 0.02^**ab**^	0.58 ± 0.02^**ab**^	0.42 ± 0.02^**b**^	0.61 ± 0.07^**a**^	= 0.009
**Cortisol,** **μd/dL**	10.33 ± 1.93	11.07 ± 2.29	8.44 ± 1.21	8.73 ± 0.75	8.26 ± 0.91	9.85 ± 0.67	8.02 ± 0.81	ns
**NEFA,** **mmol/L**	0.28 ± 0.05^**b**^	0.32 ± 0.09^**ab**^	0.48 ± 0.17^**ab**^	0.19 ± 0.02^**b**^	0.25 ± 0.03^**b**^	0.28 ± 0.03^**b**^	0.65 ± 0.10^**a**^	= 0.004
**TG, mg/dL**	16.06 ± 1.67^**ab**^	12.41 ± 1.49^**b**^	13.25 ± 1.28^**b**^	12.03 ± 1.27^**b**^	12.53 ± 0.77^**b**^	19.08 ± 0.98^**a**^	14.44 ± 0.89^**ab**^	= 0.003
**Chol-T,** **mg/dL**	261.54 ± 5.15	288.93 ± 5.20	290.29 ± 15.28	304.60 ± 18.23	289.03 ± 14.29	293.50 ± 13.51	275.04 ± 12.87	ns
**Chol-HDL, mg/dL**	104.55 ± 4.66	106.14 ± 3.57	113.03 ± 3.63	109.57 ± 5.00	103.19 ± 5.91	105,65 ± 4,03	103.18 ± 4.84	ns
**Chol-LDL,** **mg/dL**	156.98 ± 7.31	182.79 ± 3.83	177.26 ± 12.49	195.03 ± 15.19	185.83 ± 12.76	187.85 ± 10.09	171.86 ± 10.11	ns

Notes: NS: non-significative.

a p < 0,030 between pre and post-exercise at 30d for NEFA.

b p < 0,020 for hGH and p < 0,015 for NEFA between pre and post-exercise at 60d.

c p < 0,028 for hGH and p < 0,009 for NEFA between pre and post-exercise at 90d; hGH: Growth hormone; NEFA: non-esterified fatty acids; TG: triglycerides; Chol-T: total cholesterol; Chol-HDL: HDLcholesterol; Chol-LDL: LDL cholesterol.

**Table 7:** Blood count of yearling Mangalarga Marchador foals submitted to a progressive exercise protocol in a semiautomatic exerciser, supplemented with beet-pulp concentrate.

Biomarker	Experimental phase							P value
	Pre-test	30d		60d		90d		
		Pre-exercise	Post-exercise	Pre-exercise	Post-exercise	Pre-exercise	Post-exercise	
**RBC, x10**^**6**^**/μl**	11.90 ± 1.5^a^	8.82 ± 0.25^b^	8.78 ± 0.14^b^	9.38 ± 0.26b	9.38 ± 0.30^b^	9.63 ± 0.13^ab^	9.18 ± 0.17b	= 0.005
**Hb, g/dL**	7.96 ± 1.70^b^	11.25 ± 0.4^a^	11.23 ± 0.2^a^	11.70 ± 0.36^a^	11.79 ± 0.35^a^	12.54 ± 0.30^a^	11.81 ± 0.14^a^	= 0.001
**Htc, %**	44.63 ± 6.1^a^	33.01 ± 1.10^**b**^	32.99 ± 0.66^b^	35.56 ± 1.02^ab^	35.60 ± 1.03^ab^	36.59 ± 0.81^ab^	34.71 ± 0.42^ab^	= 0.016
**MCV, fl**	37.21 ± 0.68	37.43 ± 0.51	37.59 ± 0.48	37.94 ± 0.44	38.00 ± 0.39	38.01 ± 0.53	37.86 ± 0.48	ns
**MCHC, g/dL**	16.86 ± 1.19^b^	34.04 ± 0.21^a^	34.01 ± 0.12^a^	32.89 ± 0.20^a^	33.09 ± 0.1^a^	34.26 ± 0.27^a^	34.03 ± 0.22^a^	<0.001
**RDW-SD, fl**	32.27 ± 0.75	33.16 ± 0.43	33.25 ± 0.45	32.74 ± 0.54	32.78 ± 0.55	32.59 ± 0.64	32.45 ± 0.57	ns
**RDW-CV, %**	23.57 ± 0.2^**ab**^	23.64 ± 0.2^**a**^	23.33 ± 0.3^**ab**^	22.79 ± 0.2^**ab**^	22.73 ± 0.28^**ab**^	22.74 ± 0.22^**ab**^	22.60 ± 0.20^**b**^	= 0.02
**Platelets, X10**^**3**^**/μl**	189.7 ± 18.18^**ab**^	147.25 ± 10.48^**b**^	192.38 ± 11.91^**ab**^	171.38 ± 12.90^**ab**^	209.38 ± 12.51^**a**^	204.00 ± 16.50^**ab**^	227.38 ± 14.26^**a**^	<0.005
**Leukocytes, X10**^**3**^**/μl**	10.87 ± 1.37	11.32 ± 0.54	11.41 ± 0.32	12.29 ± 0.78	12.70 ± 0.46	11.85 ± 0.68	11.74 ± 0.58	ns

Note: Different upper letters in the same line indicate significant differences between moments by Tukey test (p < 0.05). Different lower letters in the same line denote significant differences between pre and post exercise for each day of evaluation by Tukey test (p < 0.05). Hb: hemoglobin concentration; Htc: hematocrit; MCV: mean corpuscular volume; MCHC: mean corpuscular hemoglobin concentration; RDW: red blood cell width distribution; SD: standard-deviation; CV: coefficient of variation.

## 4 Discussion

The hypothesis posited by the authors was confirmed in both trials. In Experiment-1, our results showed that supplementation with beet pulp concentrate increased some blood parameters without harming the health of the animals. Additionally, when analyzing the postprandial curves before and after 90 days of supplementation with the experimental concentrate, we observed an increase in the concentration of insulin and glucose, which suggests an adaptation of the digestive system during the supplementation period, possibly an increase in expression of glucose transporters [26] to absorb glucose produced during pre-cecal digestion and post-cecal fermentation of beet pulp. It is also observed that the slight reduction in the percentage of hematocrit associated with the absence of variation in plasma proteins, indicated a reduced impact on the postprandial plasmatic volume of the experimental concentrate. This information here helps to understand how the supplementation with concentrate rich in beet pulp impacts the digestive process and how this experimental concentrate can be used for different groups of horses, mainly athletic horses that are regularly supplemented with concentrate rich-grains and reduced supply of quality fiber.

Since the maximum recommended concentration of BPC did not induce any health problems and increased biomarkers associated with fat metabolism, in the case of T-Chol and HDL-chol, in addition to increasing the concentration of TPP, which is associated with better nutrition with greater protein reserves, in the group of non-exercised mares, Trial-2 was designed to evaluate the effects of a training program using a circular equicizer in a group of yearling foals fed with BPC. Thus, diet supplementation incorporating an experimental concentrate with beetroot pulp for the foals improved energy availability, both from the pre-cecal and post-cecal digestion due to the supplementation with digestible fiber present in the experiment concentrate. These processes increased the triglycerides concentrations that are used to produce NEFA, mainly in the post-exercise phase of the yearling foals after 60 days of supplementation, allowing an improvement in the athletic capacity of these foals during the exercise and the recovery phase. Moreover, this training program with BPC did not promoting the appearance of lesions in the locomotor system with OCD, which can occur in yearling foals at that age when supplemented with concentrates rich in soluble carbohydrates. Furthermore, in this Trial-2, the elevation of post-exercise hCH secretion stimulated overall anabolism with greater impact on skeletal muscles and other body tissues, resulting in adequate adaptation to the exercise program. In Trial-1, the use of BPC for 12 weeks produced changes in both carbohydrate and fat metabolism, without compromising the animals' health, since no gastrointestinal issues nor lameness were observed. These changes indicated that the BPC not only increased the availability of energy compounds, principally SCFA, but also maintained glucose and insulin levels without significant changes in fasting animals. These results were attributed to the characteristics of fibers present in the BPC, which have been reported to improve the digestive process, increase gut microbiota diversity and richness, enhance both pre-cecal and post-cecal digestion [8,10,13,[27], [28], [29]]. It was also found that, the use of BPC kept the levels of these biomarkers below those considered harmful to horses (glucose: <175 mg/dL; insulin <36 IU/mL), despite the increase in glucose and insulin levels in the post-supplementation curves [2,[30], [31], [32]]. This is important from the standpoint of horse nutrition, since it was recently found that circulatory disorders in the laminar tissue of the hoof, typically observed in laminitis, may occur when insulin levels remain high for long periods [2]. In this context, it was found that the average glucose and insulin levels in the postprandial period, on both the pre- and post-supplementation curves, did not reach those described in the literature as harmful to equine health [19]. However, there is still no clear consensus about dangerous levels, especially in the case of insulin since there are many laboratory techniques for measuring this hormone. The use of beet pulp in the feed of different types of horses has expanded in several countries. Studies on cecal-fistulated horses have shown that the increase in the proportion of digestible fiber on horse concentrate, as in beet pulp, stimulates the production of SCFA, which increases energy viability after 9 h [33]. Also, these authors comment that post-prandial concentration of glucose and insulin were impacted by the amount of fiber inclusion on hay-fed ponies [1]. In addition, the fiber quality of the BPC promotes increase in propionate and butyrate production, and these SCFA stimulate t h e production of blood fat biomarkers and improve local protection of the intestinal cells [4,13]. This confirms the importance of this type of feed to modify paradigms related to the inclusion of more digestible fiber, thereby reducing the amount of soluble carbohydrates for horses. However, it may be difficult to results with those reported in other studies, since the inclusion of beet pulp varies from 16 %, as used in our experiment, to up to 25 % as reported in other experimental protocols, depending on if the BPC is combined with other nutrients, such as oils [27,[34], [35], [36]]. The present research confirms that the inclusion of digestible fibers for athletic horses can maintain performance levels and still preserve gut health. The larger amount of fiber in the concentrate resulting from the addition of beet pulp stimulates chewing and saliva production, favoring the digestion of the compounds in feed; contrarily, diets rich in carbohydrates reduce chewing time leading to diseases in the gut [8,15]. In ruminants, chewing time increases when soybean hulls are included in feed, since they are rich in digestible fibers, favoring the production of short chain fatty acids [6]. This process may also occur in other herbivores such as horses, when they eat feeds rich in highly digestible fibers containing pectin. It has also been demonstrated, in ponies, that the digestion of beet pulp in the small intestine is high when compared with other types of products rich in digestible fibers [29]. This entire process helps raise postprandial glucose levels in animals that were fed beet pulp, reflecting the findings of our current study. This characteristic may increase the availability of glucose for glycogen production without overloading the intestines of sports horses with grains [28,37], even with the insulin levels found on the postprandial curve produced in the post-supplementation phase. An evaluation of lipid biomarkers indicated that BPC supplementation probably increased the availability of SCFA, principally propionate, thereby stimulating oxidation and the formation of lipids in liver tissue, such as cholesterol and its fractions [33,38]. This was expected since the inclusion of beet pulp in feed favors the production of acetate and propionate [1]. The mean concentrations on the triglyceride curves indicated a reduction in the concentration of this biomarker, probably due to low insulin levels, and increased lipolysis and blood lipase levels [35,39,40], forming NEFA and glycerol. In this experiment, the highest increases in insulin levels on the post-supplementation curve produced lower triglyceride concentrations, although fasting animals showed no changes in the levels of this biomarker and insulin. Animals fed exclusively with forage were able to maintain high levels of NEFA and acetate and low insulin levels over a 12-h fasting period, unlike a group that was fed with oats and fodder [39]. When horses are fed a concentrate containing 25 % of beet pulp, their triglycerides and NEFA levels decrease and insulin levels increase significantly (+3 h, 20 IU/L) [35]. However, in this study, insulin levels were about 10 IU/mL, although the amount of beet pulp added to the BPC was lower (∼16 %). Greater fiber digestibility may modify the production of nitrogen compounds and some B-complex vitamins. Several authors have inferred that beet pulp supplementation could impair nitrogen uptake, so animals that are fedsuch fibers would require greater amounts of nitrogenous compounds [27,36]. In the current experiment, only the concentration of TPP was measured, and this biomarker remained unchanged along the curve. However, the concentration of this biomarker increased (∼20 %) over the 12 weeks of supplementation, although there was no increase in hematocrit levels, indicating that this process was not due to hemoconcentration. TPPs are not only important regulators of blood homeostasis but also serve as a nutrient reservoir [41], and their elevation may also indicate increased reserves of amino acids and other compounds transported by them. Most of the hematological biomarkers were not affected by the supplementation program, except for the MCHC. However, changes in these parameters were not expected, given that the animals’ health remained normal throughout the experimental protocol.

The use of highly digestible fibers for athletic horses can be a good option when considering availability of energy sources and maintenance of gut health. Beet pulp has been shown to contain a combination of digestible fibers that provide more nutrients throughout the digestive process than other sources of fiber of similar quality [29]. The high digestibility of beet pulp resembles that of oats and soybean hulls, increasing the availability of energy compounds and favoring pre-cecal digestion, because it has available sugars, and post-cecal digestion, thus increasing the production of SCFA [[27], [28], [29]], which is important when considering athletic animals. On trial 2 we considered that yearlings in a resistance aerobic training program could have positive effects when fed BPC. After the 90-day training protocol, the animals improved their physical conditioning without comprising their welfare since both duration and speed of exercise increased (∼33 %). Regular training stimulates the general metabolism of animals to adapt to the type of exercise that is related both to its duration and intensity [42]. The processes induced by exercise are reflected in changes in blood constituents, due to a release of catecholamines and cortisol, mainly aiming to increase the supply of energy to the muscles in activity [43]. Exercise, whether acute or repetitive, is a potent secretory stimulus for growth hormone (hGH) in horses. This hormone is beneficial in the animals' post-exercise recovery, since it stimulates gluconeogenesis, glycogenolysis and lipolysis [44] favoring protein synthesis and energy availability in exercises lasting longer than 10 min [45]. In the current study the animals were submitted to an exercise program with progressive increase in speed and duration, and post-exercise elevations of hGH were detected when the animals were already two months into training, similarly, to changes in NEFA concentrations. These results are like those described by different authors and are important for post-exercise recovery [44,45]. Such responses should impact the development of early athletic capacity and, therefore, favor the athletic longevity and well-being of this group of horses. Elevation in hGH and NEFA may also be influenced by nutritional factors [46]. Secretion of hGH can be inhibited by feeding foods with a high glycemic index or rich in starch compared to those with a low glycemic index, such as concentrates rich in digestible fiber. In Trial-2, yearling foals were supplemented with a concentrate rich in beet bulp and oils, which may have influenced the concentrations of both hGH and NEFA. However, further studies are still needed on the regulatory mechanisms and possible long-term effects for the understanding of the use of nutrition to modulate the somatotropic axis in growing horses that perform stimulated physical exercises, mainly concentrates rich in digestible fibers and oils. Furthermore, it was observed that the concentrations of NEFA and triglycerides increased at the end of the experimental period, which could be explained both by the release of hGH and catecholamines, which favor lipolysis [47], increasing the availability of substrate for performing and maintaining exercise combined with the appropriate nutritional program for athlete animals with submaximal exercises. In gaited horses, NEFA concentration increased with supplementation with oil-rich concentrates and after exercise [12,48,49], indicating that the increase in the concentration of this biomarker can be obtained in animals in submaximal training regimen when supplemented with food rich in oil plus digestible fiber.

Conversely, cortisol concentrations did not change during the experimental period. These results may be correlated with the progressive activity program, where the animals gradually adapted to the exercise intensity. Cortisol concentration can be used as an index of stress in animals and as an evaluator of the impact of training, since studies report that its release is lower in trained animals when compared to untrained ones [43,45], when they are subjected to the same type of activity. In gaited horses submitted to a standard test for athletic evaluation, an absence of cortisol elevation was also observed, but with an increase in the NEFA concentration after the exercises [48]. It should also be considered that the type of training imposed, with the use of a horse walking machine, causes the physical activity to be performed in a group, just as it happens with natural exercises in pastures, which may also have contributed to the results recorded for cortisol. The proposed physical activity changed the hematological biomarkers analyzed, which are often used to assess horses’ athletic performance [49]. Hematimetric variables such as hematocrit (Htc), red cell count and hemoglobin concentration (Hb) can be used to assess the effects of both exercise and training [50,51], helping to understand the adaptations that occur during the practice of physical activity. Higher red blood cell concentrations and percentage of hematocrit observed in the pre-test of the experimental period are possibly related to a greater splenic contraction, associated with a possible lower physical conditioning. Associated with this, higher concentrations of hemoglobin and CHCM were observed at the end of the training period, suggesting an adaptation to meet the greater demand fortissue oxygen, due to the increase in metabolism due to the exercises developed in the current training program, likebserved previously in gaited horses [49].

## 5 Conclusions

These current results showed that supplementation with beet pulp concentrate, with a high content of digestible fiber, led to modifications on the studied blood biomarkers over the course of the 12 weeks of supplementation and changed postprandial curves, mainly increasing the availability of carbohydrates (glucose) and fats (cholesterol andits fractions) on Trial-1, suggesting an adaption of the digestive system to absorb more glucose and short-chain far cids produced during digestion and fermentation of the beet pulp. In Test 2, there was an improvement in the physical conditioning of yearling foals trained in a circular equine equicizer. In addition, the inclusion of BCP led to greater availability of triglycerides and NEFA during and after training, which was also accompanied by an increase in hCG concentration, which is essential for developing structures linked to exercises. These combined results suggest that the corporation of beet pulp to the concentrate, as a source of fiber and energy, could be used for young animals at the beginning of training, contributing positively to these aspects.

## CRediT authorship contribution statement

**Luzilene Araujo de Souza:** Writing – original draft. **Monica Miranda Hunka:** Data curation, Conceptualization. **Sigismundo Fassbender de Rezende Júnior:** Methodology, Investigation, Data curation, Conceptualization. **Carolina Jones Ferreira Lima da Silva:** Visualization, Validation, Methodology, Data curation, Conceptualization. **Helena Emília Cavalcanti da Costa Cordeiro Manso:** Validation, Formal analysis, Data curation, Conceptualization. **Joana Simões:** Validation, Formal analysis, Data curation, Conceptualization. **Clarisse Simões Coelho:** Writing – review & editing, Visualization. **Francesco Fazio:** Visualization, Supervision. **Francesca Aragona:** Visualization, Validation, Formal analysis, Conceptualization. **Hélio Cordeiro Manso Filho:** Software, Investigation, Data curation, Conceptualization.

## Institutional review board statement

The research project was approved by the Ethics Committee on Animal Use of the Federal.

Rural University of Pernambuco (CEUA-UFRPE), under protocol no. 44/2018 e 73/2019. Informed Consent Statement: Not applicable.

## Data availability statement

The data presented in this study are available on request from the corresponding author.

## Funding

This study was supported by funding from the UFRPE - Núcleo de Pesquisa Equina. L.A.S. and C.J.F.L.S received a partial and full scholarship from the 10.13039/501100002322Coordination for the Improvement of Higher Education Personnel (CAPES).

## Declaration of competing interest

The authors declare the following financial interests/personal relationships which may be considered as potential competing interests:

Francesco Fazio reports a relationship with Heliyon journal that includes: board membership. If there are other authors, they declare that they have no known competing financial interests or personal relationships that could have appeared to influence the work reported in this paper.
